# Measuring assessment standards in undergraduate medical programs: Development and validation of AIM tool

**DOI:** 10.12669/pjms.341.14354

**Published:** 2018

**Authors:** Madiha Sajjad, Rehan Ahmed Khan, Rahila Yasmeen

**Affiliations:** 1 Dr. Madiha Sajjad, FCPS, MHPE. Department of Pathology, Riphah International University, Rawalpindi, Pakistan; 2 Dr. Rehan Ahmed Khan, FCPS, FRCS, MHPE. Department of Surgery, Riphah International University, Rawalpindi, Pakistan; 3 Dr. Rahila Yasmeen, MHPE. Department of Medical Education, Riphah International University, Rawalpindi, Pakistan

**Keywords:** Faculty perceptions, Assessment, Quality, Standards, Tool, Development, Validation

## Abstract

**Objective:**

To develop a tool to evaluate faculty perceptions of assessment quality in an undergraduate medical program.

**Methods:**

The Assessment Implementation Measure (AIM) tool was developed by a mixed method approach. A preliminary questionnaire developed through literature review was submitted to a panel of 10 medical education experts for a three-round ‘Modified Delphi technique'. Panel agreement of > 75% was considered the criterion for inclusion of items in the questionnaire. Cognitive pre-testing of five faculty members was conducted. Pilot study was done with 30 randomly selected faculty members. Content validity index (CVI) was calculated for individual items (I-CVI) and composite scale (S-CVI). Cronbach's alpha was calculated to determine the internal consistency reliability of the tool.

**Results:**

The final AIM tool had 30 items after the Delphi process. S-CVI was 0.98 with the S-CVI/Avg method and 0.86 by S-CVI/UA method, suggesting good content validity. Cut-off value of < 0.9 I-CVI was taken as criterion for item deletion. Cognitive pre-testing revealed good item interpretation. Cronbach's alpha calculated for the AIM was 0.9, whereas Cronbach's alpha for the four domains ranged from 0.67 to 0.80.

**Conclusions:**

‘AIM' is a relevant and useful instrument with good content validity and reliability of results, and may be used to evaluate the teachers´ perceptions about assessment quality.

## INTRODUCTION

Medical schools face the challenge of developing and implementing quality assessment programs that are acceptable to accrediting bodies and society at large. Evaluating the assessment system at intervals assures that assessments remain effective and up-to-date.^1^,^2^ Several instruments are available in literature to evaluate educational environment for both students^1^ and teachers^3^ at undergraduate institutes as well as in clinical environment.^4^ However based on current literature, no validated survey instrument has been identified in an undergraduate medical context to reliably evaluate the quality of assessment. Perceptions about assessment process in an institute can provide important information about gaps between accepted standards and the implemented assessment practices.^5^ Quality assurance in assessment requires involvement of the entire institutional team especially a faculty well acquainted and engaged in the culture of student assessment.^6^ However, despite the important role of faculty in successful implementation of assessments, little focus is given in the medical education literature about medical teachers' perceptions about assessment in institutes as an indicator of the quality of student assessment.

This study aims to develop and validate a tool in order to evaluate the quality of assessment practices and for institutional self-evaluation to inform, guide and improve assessment quality.

## METHODS

A mixed methods study design was used with sequential qualitative and quantitative components in the following four-stage process for developing and validating the questionnaire:

### 1. Review of literature and preliminary questionnaire item development

Quality indicators of assessment were identified from literature search based on the quality standards provided by multiple sources such as the WFME document^7^, LCME^8^, CACMS^9^ and students' perception questionnaire^1^ etc. A preliminary draft questionnaire of 34 items was prepared for further amendments through the Delphi technique.

### 2. Modified Delphi technique for consensus development on questionnaire items and content validation of the AIM tool

A 3 round modified Delphi approach was used in which 18 medical education experts having a diploma/degree in medical education and working in undergraduate medical institutes, were invited to participate through email. In round one, the panellists were asked to grade ‘relevance' of items, on a five point Likert scale. Percentage responses and median scores for each item were calculated. For round 2, items were added or amended based on results and the questionnaire was resent to the panelists. The panelists were instructed to either accept the suggested items, reject with reason and propose modifications where considered necessary. Panel agreement of > 75% on each statement was considered the criterion for inclusion of items in the subsequent round. In round 3, the panel was asked to indicate relevance of each item on a 4-point Likert scale for final inclusion of the item into the questionnaire. The panel was also provided with a list of four domains, and the item statements allocated to it. They were requested to indicate agreement to the domain allotted for the statements and if not in agreement, to reallocate the statement to their preferred choice of domain. Panel agreement of > 75% was considered as inclusion criteria of item in the given domain.

Content validity index (CVI) for the individual items (I-CVI) and of the scale (S-CVI) was calculated using the ratings of item relevance by content experts in the last round.

### 3. Cognitive pretesting to check for faculty understanding

Five faculty members were selected for cognitive pretesting through convenience sampling method. Individual interviews were conducted through ‘concurrent verbal probing method'.^10^ The 4 cognitive validity criteria used were: ‘correct item interpretation, comprehensible explanation, answer choice compatibility with interpretation, and overall item cognition' across the 5 participants.^11^,^12^

### 4. Pilot study on a sample of faculty to establish the reliability of the tool

**Fig. 1:**
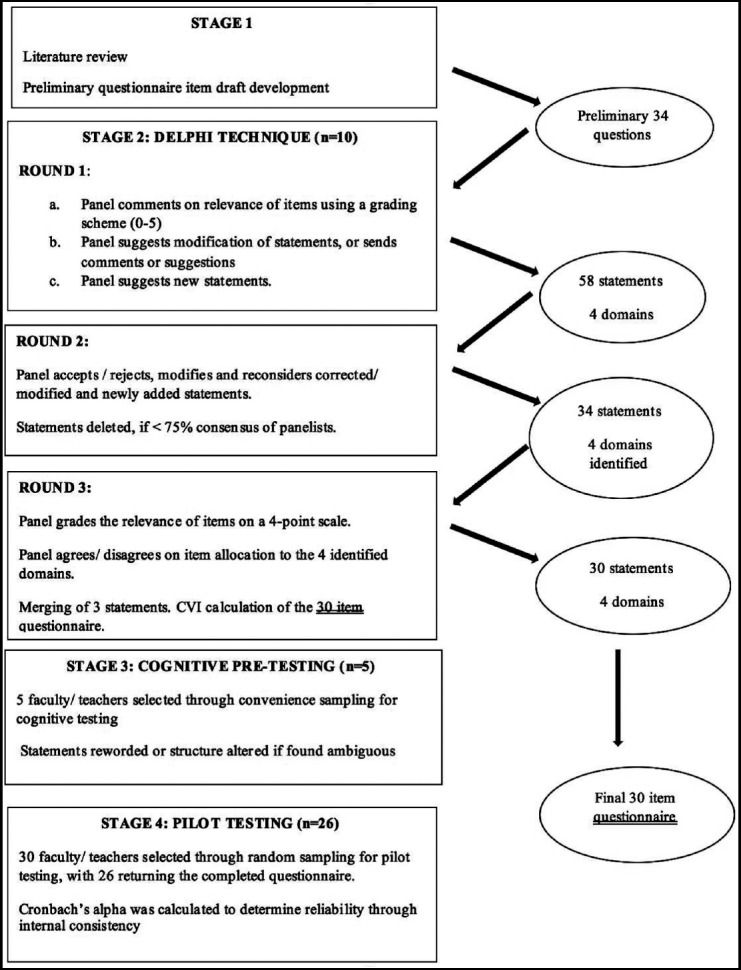
Methodology flowchart.

The final 30 item questionnaire was given to 30 randomly selected participants, from both the basic and clinical sciences faculty, in order to determine the reliability of the final developed AIM tool. The summary of methodology is given in Fig.1.

## RESULTS

### Quality indicators of assessment identified after literature review

The 13 quality indicators identified from literature search were: Assessment principles regarding criteria for setting pass marks, grade boundaries and allowed retakes; conflict of interest policy; assessment methods; Assessment of the learning domains; number and type of assessment per educational objective; number and type of assessment per instructional method; students meeting each educational outcome; students right of appeal against assessment results; feedback received by students: frequency, timing and nature; formative and summative assessments; assessment utility: validity and reliability; integrated learning; use of external examiners. Based on these indicators, a preliminary 34 items questionnaire was developed.

### Delphi Process results

In Round 1, 10 out of 18 (n=56%) panelists returned the completed preliminary questionnaire. Based on the consensus results, perceived double barreled statements were simplified and new items were added for the Round two questionnaire forming a total of 58 items. After Round 2, 24 statements were rejected and two were merged, making a total of 34 statements for the round 3 questionnaire. After Round three, two items were deleted and three were merged. The final tool consisted of 30 items under four suggested domains as given in Table-I.

**Table-I:** AIM tool showing subdomains with allocated items.

*Assessment policies*	
1	The medical school has a clearly defined assessment policy.
2	I have been oriented about the assessment policy in my college.
3	The procedures used for assessment of students are clearly laid down in assessment policy.
4	The criteria of student progression to next class are clearly documented.
5	The number of allowed exam retakes are clearly documented.
14	A system of appeal against assessment results is in place.
15	Assessments are open to scrutiny by external experts.
27	Standard setting is used to decide Pass/fail criteria before each individual summative assessment.
*Assessment methods*	
6	The assessment methods used to assess knowledge component of course are appropriate for assessing the cognitive domain.
7	The assessment methods used to assess skill component of course are appropriate for assessing the psychomotor domain.
8	The assessment methods used to assess behavior component of course are appropriate for assessing attitude domain.
9	An appropriate weightage is given to knowledge, skills and attitude domains in assessments.
11	The assessment methods used are feasible.
16	Use of new assessment methods is encouraged, where appropriate.
23	Clear blueprints (table of specifications) are provided for each assessment.
25	Checklists or rubrics for performance assessments are clearly documented.
*Purpose of assessment*	
17	The assessment system promotes student learning
18	Formative assessments are done at appropriate points during the curriculum to guide student learning.
19	There is an appropriate mix of formative and summative assessments.
20	The assessments encourage integrated learning by the students.
21	Feedback is given to students promptly after an assessment.
*Assessment quality measures*	
10	Assessment system ensures that all assessments are conducted fairly
12	Adequate resources are provided for all assessments.
13	There is an adequate role of external examiners in summative examination.
22	Teachers are trained to provide feedback to students
24	Assessments adequately represent the exam blueprints (table of specifications).
26	There is an item bank which teachers contribute to and use for preparing exams.
28	Post examination item analysis is regularly conducted for summative assessments.
29	Post examination item analysis results are communicated to concerned departments.
30	Faculty development workshops are regularly conducted on various aspects of assessment.

### Content validity index (CVI) calculation

Content Validity index of individual items (I-CVI) as well as the scale (S-CVI) was calculated. S-CVI was calculated with two methods, S-CVI/Avg and S-CVI/U, and is shown in Table-II.

**Table-II:**
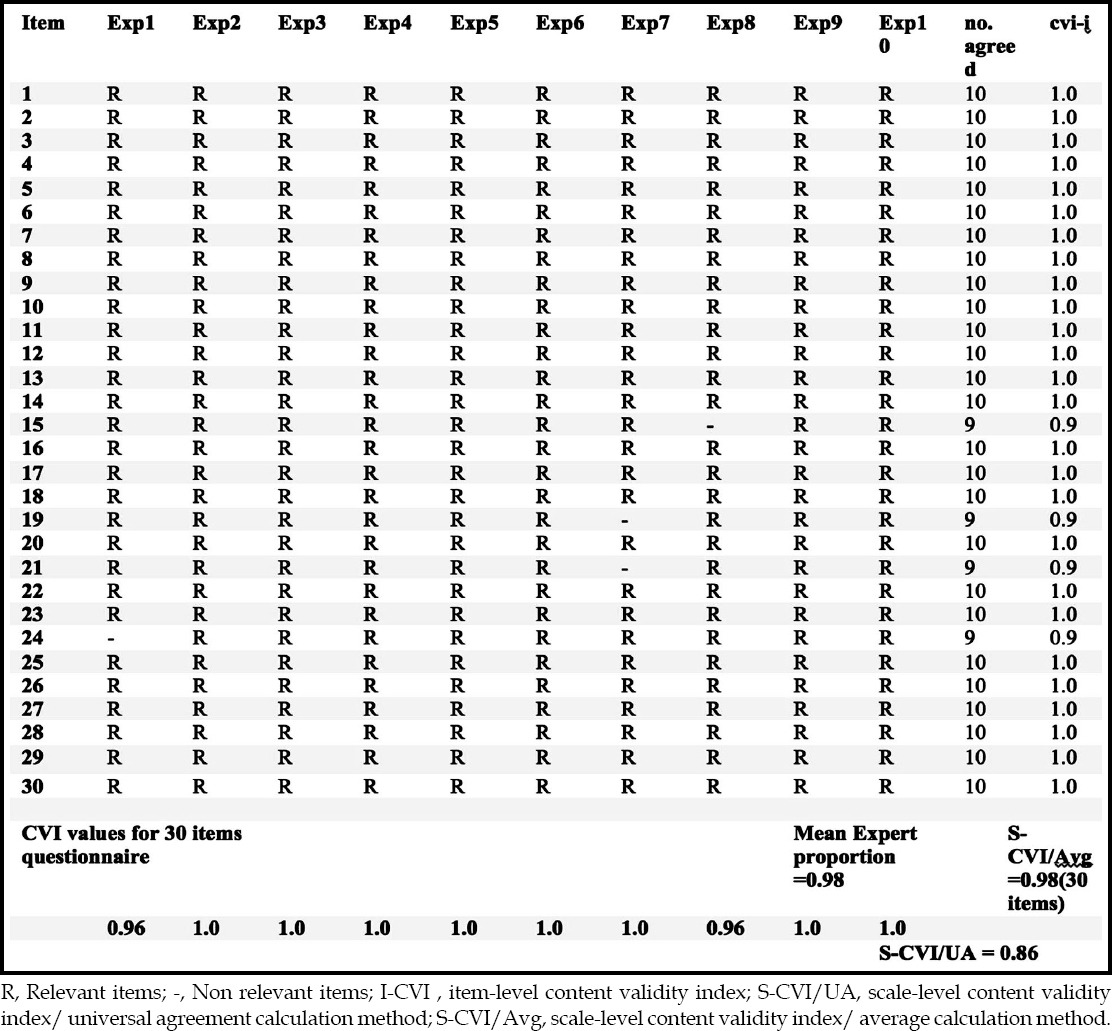
Content validity index calculation.

### Cognitive pre-testing

Cognitive pretesting of the questionnaire resulted in minor adjustments to the statements. A few technical terms like ‘standard- setting' was considered ambiguous by some participants who considered ‘pass/fail criteria' as the more relevant phrase for cognition.

### Pilot testing for reliability of AIM tool

The response rate was 86% (n=26/30). Data was entered into SPSS 20, to calculate the reliability of the scale and its assigned domains. The calculated internal consistency for the composite score and all domains are given in Table-III.

**Table-III:** Cronbach's alpha for domains and full AIM tool.

Domains	No. of items	Cronbach's alpha
Assessment policies	8	0.78
Assessment methods	8	0.80
The purpose of assessment	5	0.67
The quality measures in assessment	9	0.73
Full questionnaire scale	30	0.915

## DISCUSSION

The AIM tool was developed through an assorted methodology. A ‘modified Delphi technique' was used rather than a focus group discussion forum, to assure respondent anonymity and to reduce unnecessary communication deterring from focussing on problem solving.^13^

For Delphi result analysis, the acceptable level of consensus needs to be determined beforehand. Different acceptable agreement levels between panelists are reported ranging from 51%-80%, or specified by stability of the response through the iterative process.^3^ In our study, percentage responses along with the medians and ranges were calculated in Round 1. For subsequent rounds, we pre-specified a panel agreement of more than 75% as a criterion for achievement of consensus. For round 3 final analysis, content validity index (CVI) of the individual items as well as that of the whole scale was also calculated using the ratings of item relevance by content experts. Good content validity of items is considered with I-CVIs of 1.00 with 3 to 5 experts and a minimum I-CVI of 0.78 with 6 to 10 experts. For scale level CVI, 0.90 or higher index is desired using the average calculating method and at least 0.80 is required using the universal agreement method, as it is more stringent in its approach.^14^ In our study all the results were well above the desired range.

For ‘Cognitive pre-testing' a respondent number of 10-30 or as few as 5-6 for a small scale research design, is considered sufficient.^12^ We interviewed five faculty members using the ‘Concurrent verbal probing method' as it eliminates the recall bias.^10^

To determine face validity, reliability and feasibility in certain large scale studies, pilot study is recommended.^1^,^3^ For initial scale development, 30 representative participants from the population of interest is considered a reasonable minimum sample for pilot study, with a range from 25-40.^15^ In our study, we selected 30 participants from the faculty. Cronbach's alpha was calculated for internal consistency of the tool and was calculated to be 0.9. The reported acceptable values of alpha, range from 0.70 to 0.95.^16^

### Limitations

Construct validity could not be established because of small sample size.

## CONCLUSION

The Assessment Implementation Measure (AIM) is a relevant and useful instrument to assess quality of assessment in undergraduate medical institutes. Further studies are needed for validation of AIM tool in variable contexts as well as its psychometric exploratory and confirmatory factor analyses.

### Authors' Contribution

***MS:*** Designed, did data collection, statistical analysis, manuscript drafting and editing.

***RAK:*** Conceived, designed, statistical analysis, editing and critical review of manuscript.

***RY:*** Did critical review and editing.
